# Supplementary material for: Transspinal stimulation preceding assisted step training reorganizes neuronal excitability and function of inhibitory networks in spinal cord injury: A randomized controlled trial
Source: medRxiv. 2025 Jun 12:2025.06.11.25329338. Preprint. [Version 1] doi: 10.1101/2025.06.11.25329338 (PMC12204436; doi:10.1101/2025.06.11.25329338)
Supplement: 1 [file NIHPP2025.06.11.25329338V1-supplement-1.pdf]

medRxiv preprint doi: https://doi.org/10.1101/2025.06.11.25329338; this version posted June 12, 2025. The copyright holder has placed this preprint (which was not certified by peer review) in the Public Domain. It is no longer restricted by copyright. Anyone can legally share, reuse, remix, or adapt this material for any purpose without crediting the original authors.

# Supplementary Figure 1

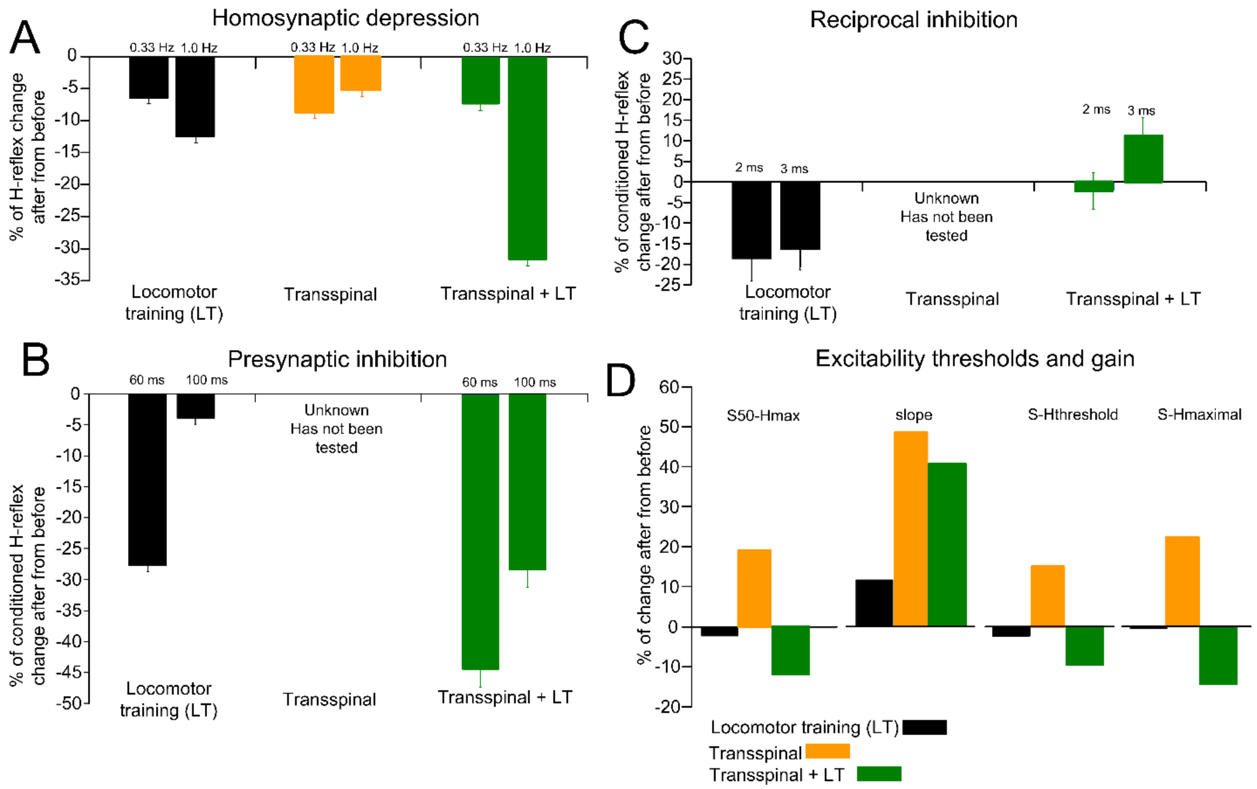


**Figure 1S. Reorganization of spinal inhibitory circuits and excitability for different interventions.** The percentage of changes after intervention from baseline **(A)** low-frequency dependent depression estimated from the soleus H-reflex amplitude evoked at 0.33 and 0.1 Hz, **(B)** presynaptic inhibition estimated by the soleus H-reflex depression by antagonistic peroneal nerve stimulation at 60 and 100 ms C-T intervals, **(C)** reciprocal inhibition estimated by the soleus H-reflex depression by antagonistic peroneal nerve stimulation at 2 and 3 ms C-T intervals, and **(D)** excitability thresholds and gain estimated from the soleus H-reflex recruitment curve are shown for clinical trials that administered locomotor training (LT) alone, transspinal stimulation alone at rest, and transspinal stimulation delivered during standing before LT within the same session (this study). It is apparent that transspinal stimulation + LT nearly doubles the homosynaptic depression and presynaptic inhibition exerted on soleus Ia afferents, while the stimuli corresponding to 50% Hmax, H-threshold, and maximal H-reflex tend to have a similar adaptation between LT and transspinal + LT trials. Only the slope was increased in a similar manner in all three different interventions. Data adopted and re-analyzed from the current study and from Knikou and Mummidisetty 2014, Smith et al. 2015, Knikou et al. 2015a, Knikou and Murray 2019.
